# Biofilm Control Strategies: Engaging with the Public

**DOI:** 10.3390/antibiotics9080465

**Published:** 2020-07-30

**Authors:** Joanna Verran, Sarah Jackson, Antony Scimone, Peter Kelly, James Redfern

**Affiliations:** 1 Department of Life Sciences, Faculty of Science and Engineering, Manchester Metropolitan University, Manchester M1 5GD, UK; S.L.Jackson10@stu.mmu.ac.uk (S.J.); T.Scimone@mmu.ac.uk (A.S.); 2 Surface Engineering Group, Faculty of Science and Engineering, Manchester Metropolitan University, Manchester M1 5GD, UK; peter.kelly@mmu.ac.uk; 3 Department of Natural Sciences, Faculty of Science and Engineering, Manchester Metropolitan University, Manchester M1 5GD, UK; james.redfern.88@gmail.com

**Keywords:** Biofilm, Public Engagement, Outreach, Control Strategies, Oral Biofilm

## Abstract

There are few peer-reviewed publications about public engagement with science that are written by microbiologists; those that exist tend to be a narrative of an event rather than a hypothesis-driven investigation. However, it is relatively easy for experienced scientists to use a scientific method in their approach to public engagement. This short communication describes three public engagement activities hosted by the authors, focused on biofilm control: hand hygiene, plaque control and an externally applied antimicrobial coating. In each case, audience engagement was assessed using quantitative and/or qualitative methods. A critical evaluation of the findings enabled the construction of a public engagement ‘tick list’ for future events that would enable a hypothesis-driven approach with more effective communication activities and more robust evaluation.

## 1. Introduction

It is increasingly being recognised by ‘experts’ that science literacy is of key importance for the public [1]. At a time where antimicrobial resistance (AMR) continues to pose significant public health threats (or indeed, at a time of a global pandemic), an understanding of statistics, epidemiology and microbiology is even more desirable. As a subject, microbiology offers many topics with which we can engage non-experts, such as microbial diversity (including fungi, algae, protozoa and viruses as well as bacteria), beneficial microbes (for example, probiotics, fermented foods, the human microbiome), and messages that can influence behaviour in a positive manner (including vaccination, hand hygiene, antimicrobial stewardship) [2,3,4].

Biofilms (an assemblage of microbial cells that are irreversibly associated with a surface—not removed by gentle rinsing—and enclosed in a matrix of primarily polysaccharide material [5]) are of great importance to microbiologists, but also to many other professionals (such as engineers, biocide manufacturers, architects), and are found in a variety of environments (water distribution systems, industrial processing, hospitals). Biofilm research is multi-disciplinary, extensive and significant, with many applications. There are several research centres which focus on biofilm, such as the US-based Centre for Biofilm Engineering (http://www.biofilm.montana.edu/) and the UK-centred National Biofilm Innovation Centre (https://www.biofilms.ac.uk/), and conferences about biofilm are regular and not uncommon. Some individual researchers, research groups and research centres are keen to engage with external public audiences through outreach activities, although evidence of such activities (websites, articles, learning materials and other peer-reviewed outputs) is not easy to find. But why do we want the public to know about biofilms? And what does the ‘public’ need to know about biofilms? How will we know if our activity has been effective? How can you identify good practice? How can you share success?

Science communication/public engagement can be seen as an emerging discipline, particularly for those scientists who have begun to question the effectiveness of their public engagement work. Evaluation of effectiveness using both quantitative and qualitative methods (‘mixed methods’) is strongly supported by education researchers [6,7], enabling the assessment of both reach (i.e., numbers) and impact (change in attitudes, perception). There are few peer-reviewed publications on the topic that are written by microbiologists: those that exist tend to be a narrative of an event rather than a hypothesis-driven investigation with appropriate evaluation. However, it is relatively easy for experienced scientists to use a scientific method in their approach to public engagement. This short communication describes three different biofilm-related public engagement activities hosted by the authors, who used lessons learned to develop a tick list for future events to enable more effective communication activities with more robust evaluation. 

### 1.1. Activity One: ’Now Wash Your Hands’ 

**Figure 1:**
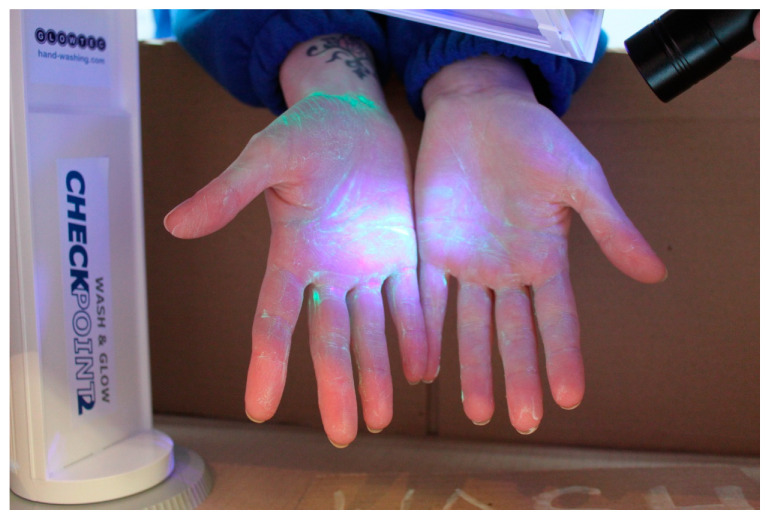
Activity one: ’Now wash your hands’: the audience engaged in hands-on activities focusing on the topic of hand hygiene. Here, a participant’s hands can be seen during the use of the UV glow gel.

**Figure 2:**
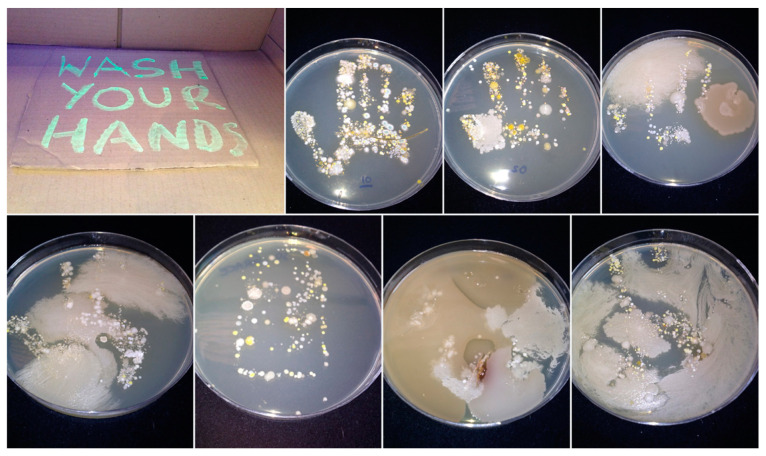
Example of the images uploaded to the Flickr page following the ‘Now Wash Your Hands’ event. Each image represents the handprint of one participant, revealing the range of microorganisms present on the hand.

‘Now wash your hands’ was developed as part of a University faculty family fun day during National Science and Engineering Week/Healthcare Science Week in the UK. The aim was to raise awareness of effective handwashing, whilst also engaging the participants in a discussion about the skin microbiome/biofilm. This event guarantees an audience of predominantly families who are likely to have an existing interest in science. Hand hygiene activities are well established as interactive learning activities with demonstrable public health impact (for example, as an intervention in reducing the spread of coronavirus [6]). In this activity, demonstrators (academic staff and student volunteers) engaged audiences to demonstrate surface contamination and effective handwashing (Figure 1). Thus, visitors at this activity (in a walkway area) had their hands ‘contaminated’ with a UV hand gel (www.hand-washing.com). This kit uses a fluorescent dye and ultraviolet light to illustrate the transmission of ‘germs’ from hands to other surfaces (and vice versa) and the importance of handwashing. In addition, the participants were invited to press their hands onto large agar plates for subsequent incubation to reveal the culturable microorganisms present on their skin. Of course, they were unable to see the results of this work until after incubation, thus images of plates pre-inoculated with microorganisms present on hands and mobile phones [7] were available to view, and post-incubation images of their own plates were uploaded to Flickr, a social media site that hosts images (http://tinyurl.com/howcleanareyourhands, Figure 2). Within a week from results going online, almost 100 downloads were recorded (the participants were provided with a card/web address), equivalent to the number of plates inoculated. From this, we deduced that visitors demonstrated interest and engagement with the activity. Throughout the activity, conversations were ongoing. It was unfortunate that these interactions were not noted in some form: informal observations revealed points of interest from the participants such as their inability to clean hands effectively (especially the adults!) and amazement at the mobile phone contamination. The handprint technique has been used as an engagement tool for other events, such as an art installation called ‘Hands across the cultures’ for registrants to a qualitative research conference and as part of the ‘bioselfies’ project (https://blogs.bl.uk/science/2020/02/introducing-bio-selfies-11-february-2020.html) initiated by the University of Salford. Flickr has been used for other events that require incubation of plates [8,9], and download numbers have on occasion exceeded the number of images posted, showing that the participants may have been sharing the findings with others. The fluorescent hand technique was used to illustrate person-to-person transmission by handshaking prior to a screening of the movie *Contagion* (directed by Soderbergh, 2011). One person ‘contaminated’ his/her hands, shook the hand of their neighbour, who shook her/his neighbour’s hand and so on. Thus, the passing-on of fluorescence was used to illustrate the transmission of infection through poor hand hygiene, reinforcing the message as to how the movie pandemic was initiated (hand contact). 

Hand hygiene activities are common in microbiology engagement, the aim of the activity being primarily to inform, and hopefully to change, participants’ behaviour so that effective handwashing techniques are employed. Explanation regarding the presence or importance of the skin microbiome/biofilm are likely rare (especially if the results are not available until a later date): the activity is inevitably more focused on the removal of temporary contaminants and on the importance of good handwashing. Some discussion could take place regarding the hygiene-versus-cleanliness hypothesis [10,11]. The Flickr method used for posting images and monitoring downloads at least gives an indication of interest, but much more could be made of this activity. It would also be interesting to know if the ‘good handwashing’ messages are retained and employed in the future. However, longitudinal studies are rare in this type of public engagement, probably because of the significant advanced planning required in terms of gaining approval for personal data access (e.g., emails) and also because only short-term awareness raising tends to be the primary aim of the activity. 

### 1.2. Activity Two: Plaque Attack!

The plaque biofilm is one of the best-known medical biofilms [12,13], and oral hygiene advertising frequently provides cartoons of plaque being removed to demonstrate the effectiveness of a paste, mouthwash or brush. It is known that good toothbrushing helps to remove plaque [14] and should be carried out regularly. Different dentifrices claim varying activities, but virtually all formulations include fluoride (to ‘strengthen the teeth’) [15], and many contain antimicrobial agents (to reduce the number of microorganisms, with claims around gum health) [16].

**Figure 3:**
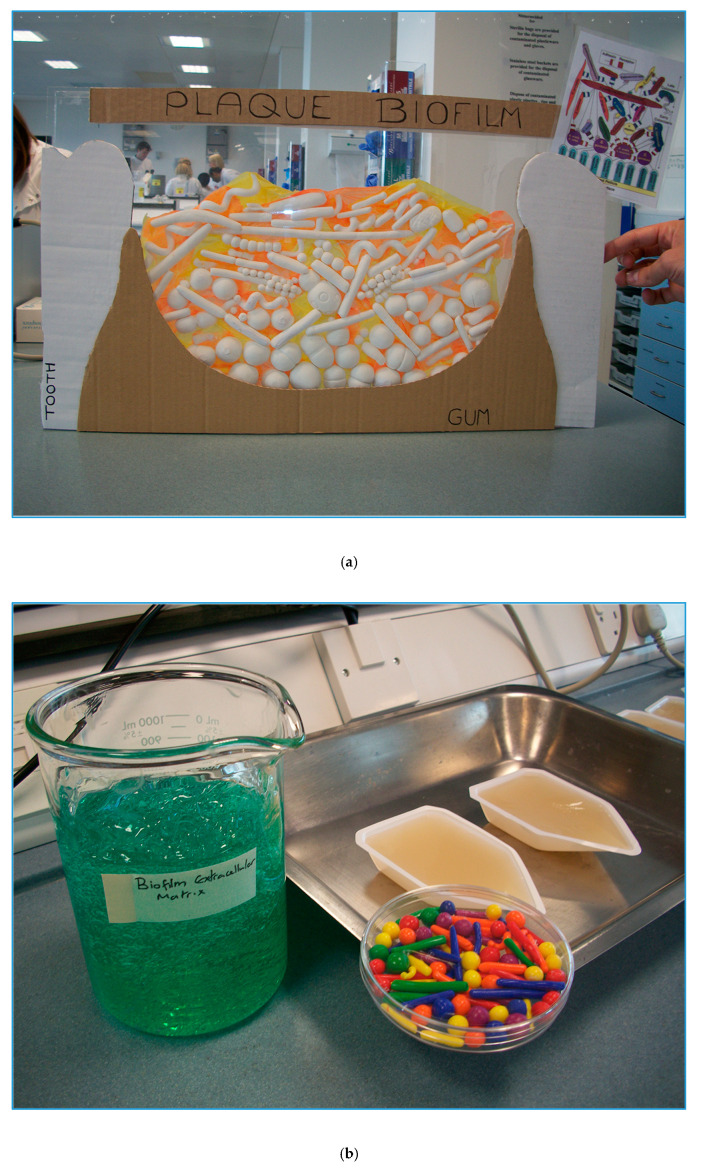
(**a/top**) Participants at the ‘Plaque attack!’ event were encouraged to create their own oral bacteria flora from modelling clay, which was assembled into the oral biofilm representation here shown. (**b/bottom**) Participants were encouraged to ‘destroy a biofilm’ by removing bacteria (coloured plastic pieces) encased in biofilm extracellular matrix (hair gel) with a spray bottle filled with water.

**Figure 4:**
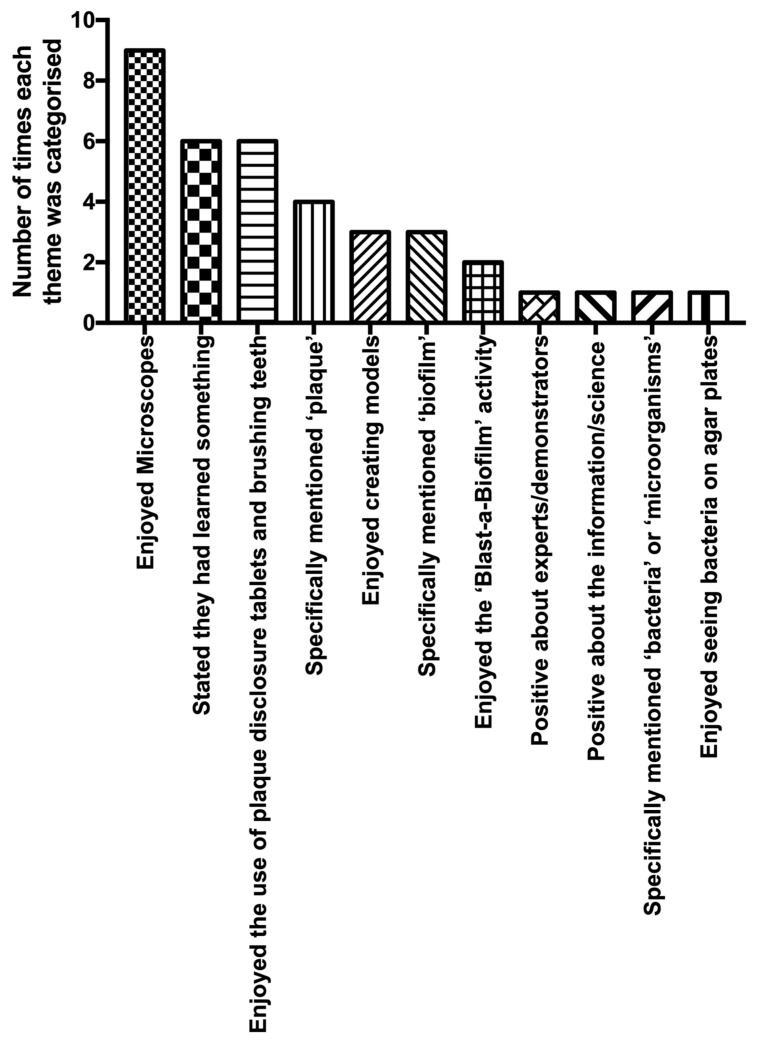
Themes identified from ‘Plaque attack!’ feedback. There was a total of 19 comments that were coded based on their focus—with each comment possibly being coded into more than one category.

‘Plaque attack!’ was a laboratory-based activity designed for children and their parents, taking place during Manchester Science Festival’s family fun day at Manchester Metropolitan University. The aim of the event was to encourage good oral hygiene but also to captivate visitors with the components of the plaque biofilm as well as the laboratory and its equipment. Being time-consuming and space-limited, the participants had to register for the event, were limited to 3 groups of 20 participants, be escorted to the laboratory, provided with appropriate clothing and instruction and supervised at all times. Oral microbiology is a key research area in our laboratories, and the delivery team thought it would be valuable for visitors to encounter activity in a working (teaching) laboratory. The delivery team comprised PhD students, technical staff and an academic. Several activities were conducted as part of a ‘round-robin’ activity: sampling plaque (microscopy demonstration and take-home photo [ZIP Mobile Printer, Polaroid]); disclosing plaque (using commercially available disclosing tablets), with photographs taken before and after cleaning teeth (in a wash area adjacent to the laboratory); looking at cultures of oral bacteria on agar plates; investigating biofilm structure/building a biofilm (using ‘Model Magic’ [Crayola Bedford UK], a white air-drying modelling clay) (Figure 3a); and destroying a biofilm (using a water pistol to remove plaque (whose microorganisms were pre-constructed from Fimo, a multi-coloured clay which can be hardened in the oven [www.staedtler.com]) hampered by plaque matrix (a translucent hair gel) [17] (Figure 3b). The participants were provided with a basic information sheet on plaque and oral hygiene, onto which they could attach their Polaroid images. They were also given a bag containing complimentary toothbrush and toothpaste (courtesy of Unilever [www.unilever.co.uk]). At the end of the activity, they were asked for free text feedback on what they thought of the event, and the information was coded into categories to allow for comparison [18,19] (Figure 4). The participants were particularly engrossed in the microscopy demonstration, being able to see their own plaque at high magnification. They also clearly had fun ‘destroying’ the biofilm but were less interested in the more passive/less exciting activity (agar plates demonstration, building a biofilm). The free text provided by the participants (allowing more thorough insight compared to multiple-choice or leading questions such as ‘give three things you have learned’, or ‘smiley face/sad face’ evaluations [18,20]) gave valuable qualitative information that was used to inform subsequent activities. 

### 1.3. Activity Three: A Photocatalytic Wall 

Our research into titanium dioxide coatings included a range of laboratory-based studies that compared different titanium dioxide concentrations in paint formulations [21]. The work described in this paper was to see whether the effect of a photocatalyst in paint could be detected by the human eye. Thus, as part of a PhD project investigating the activity of photocatalytic surfaces, one of the external walls of the University was used to illustrate the effectiveness of titanium dioxide paints in terms of self-cleaning and reduction of the formation of biofilm on the wall material. Photocatalytic material such as titanium dioxide can exhibit self-cleaning, anti-fouling and antimicrobial properties in the presence of light, which makes these materials excellent candidates for incorporation into urban buildings and infrastructure [22,23,24]. The self-cleaning properties stem from their superhydrophilic nature—as, for instance, that of a liquid (e.g., rain) rolling off the surface of a continuous body. This sheeting carries away dirt and debris, cleaning the surface in the process—as seen in the Sydney Opera House [25]. Thus, biofilm formation on the surface is delayed or prevented.

In our study, the wall, comprising concrete panels (smaller panels 190 cm x 76 cm, larger panels 406 cm x 76 cm) on a 1970s University building, was west-facing (location on Chester Street, Manchester, UK M1 5GD). Six of the panels were painted with a siloxane external paint formulation that contained or lacked the photoactive pigment (kindly provided by Tronox, www.tronox.com). Our aim was to inform the passing public about our research (an interpretation panel was affixed to the wall), and on occasion, we encouraged passers-by to participate in a longitudinal subjective assessment of the impact of titanium dioxide-containing paint on the perceived cleanliness of the panel. This engagement activity was done directly by interview and indirectly using photographs at specific times over a 44-month period.

**Figure 5:**
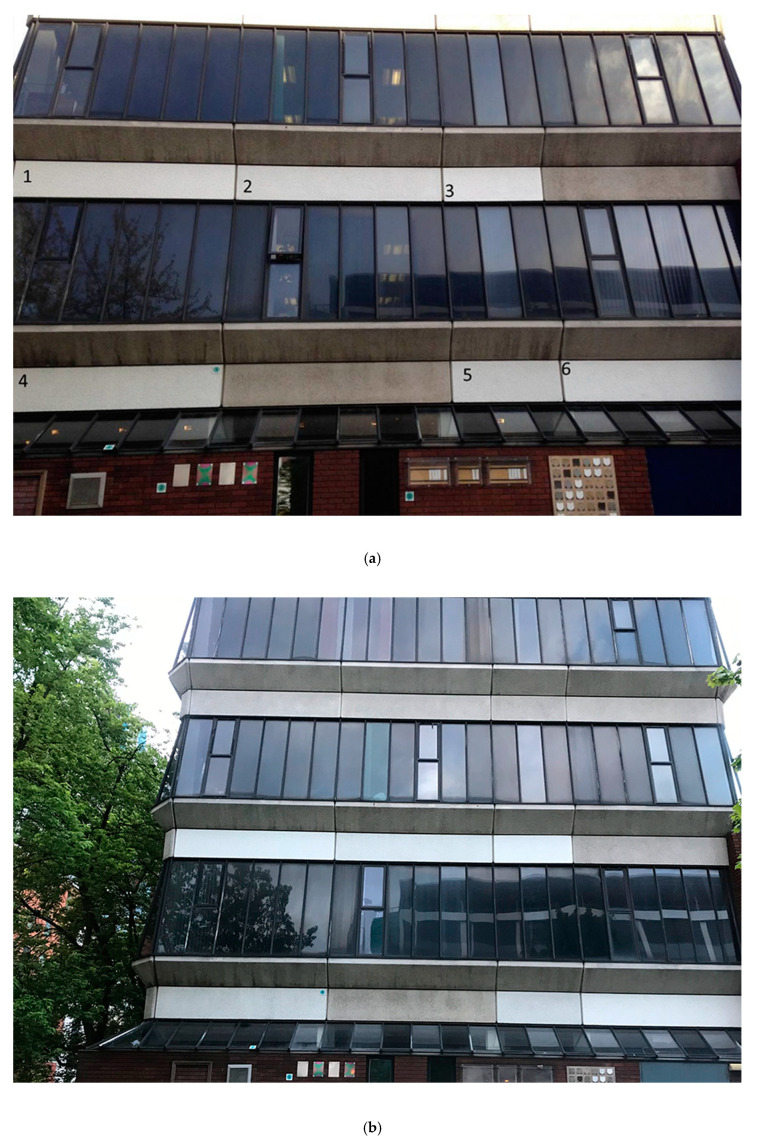
Images of the wall at Manchester Metropolitan University used in the study of photocatalytic paint (panels labelled 1–6). Panels 1, 3 and 6 were painted with photocatalytic paint, whilst panels 2, 4 and 5 were painted with paint that did not contain the photocatalytic agent. The image on the top (**a**) was taken in 2014, eight months following the application of the paint: whiteness/brightness difference between the two paint types is hard to distinguish. The lower image (**b**) was taken six years later (2020); panels painted with photocatalytic paint are visibly brighter compared to control paint panels.

Initially there was no apparent difference in the brightness of the painted panels (Figure 5a). Members of the public attending a Manchester Science Festival event (October 2014) were asked to rank the painted panels in order of cleanliness/whiteness, with 1 being most clean, and 6 being least clean (n = 18). The experiment was also conducted via a social media platform (Facebook), with participants asked to assess whiteness using photographs (n = 48). The direct assessment was repeated after three years (n = 21). In all cases, the participants ranked two or three of the photocatalytic panels as the ‘whitest’. In 2014, around 60% of the participants selected the three photocatalytic panels correctly. In 2017, this figure rose to 78%. After six years, the test-paint panels appeared whiter than the control panels (Figure 5b, May 2020).

The presence of the wall with its accompanying information panel at the side of the University Science and Engineering building provided a useful pointer to introduce visitors to some of the research ongoing in the faculty. The use of the public to assess the cleanliness of the wall proved unnecessary within a few months, when the impact of the test paint was apparent. The fact that almost all participants could discriminate between the panels after less than 12 months was also of interest. This approach might therefore be useful in the future for the assessment of test formulations.

## 2. Discussion

Much was learned from each event (as noted above), particularly through observation, in terms of what components participants like and engage with when discussing biofilm. In addition, quantitative evidence of engagement was derived from the ’Now wash your hands’ event; qualitative evidence of enjoyment and engagement was obtained from ‘Plaque attack’, and the potential for acquisition of research data was indicated by the photocatalytic wall activity. These various outcomes informed how subsequent events for the public would take place, with more focus on design, delivery and evaluation. 

More recently, there has been increasing effort to ensure that these criteria for effective public engagement are met. Microbiology has a particularly dynamic approach to public engagement, and many teams are now publishing the outcomes of their public engagement research in peer-reviewed journals, magazines or online. Yet, in a review of public engagement activity around AMR, a rich bedrock of activity was found only through personal contacts and communication rather than through a literature search [4]. It is even more important when talking to audiences about biofilms that intended messages are clear. Thus, we describe in Table 1 the planning of a hypothetical public engagement event designed to inform a large number of adults about biofilm and AMR. Our focus was on the combination of the two phenomena, which occurs, for example, when biofilms on medical devices present increased resistance to antibiotics [26]. In order to address this combined effect, it was first necessary to define the two phenomena separately. We particularly wished to avoid intrusive aspects of evaluation, relying instead on observation and other (subjective and objective) indicators from participants. We hope that this checklist may be useful for others who might wish to engage audiences with their biofilm/antibiotic research.

The National Biofilm Information Centre has recognised the importance of public engagement and is providing a hub for the dissemination of biofilm-focused outreach and engagement activities, which will enable, over time, ideas, expertise and outcomes to be shared and developed, in order to improve the effectiveness of engagement encounters for scientists and their audiences alike. We hope that our experiences in the area are of interest in this context.

## 3. Conclusions

Public engagement activities can be designed with clear aims that enable effective evaluation using both quantitative and qualitative methods. This is particularly important for complex phenomena such as biofilms and AMR. 

## Figures and Tables

**Table 1:** Checklist for public engagement events, with accompanying information detailing planning for a proposed event focusing on antimicrobial resistance (AMR) and biofilms.

	General Considerations	Example
Event	Audience, venue, time, date, numbers	Adults, evening, 3 hours, audience around 500 participants, drop-in marketplace format
Topic	Theme	AMR and biofilms
Aim/hypothesis		Aim: To engage the audience with the biofilm phenomenon and its relationship with AMR Hypothesis: That the audience will leave the event with an increased awareness of AMR and some knowledge of biofilms
The message	What specific message(s)	About AMR What biofilms are Why biofilms are important with regard to AMR
What will be happening	Focus activities on key messages, encourage active participation and engagement	Welcome table (guide to activities) Bitesize quotes about AMR on a pop-up stand iPad questionnaire used to lead discussion about AMR Screening of the film ‘Catch’ [4] Swabbing face/nasal area (anonymous) Rolling images of biofilm Discussion about biofilm Building biofilm using Bunch’ems (www.bunchems.com) Sign up to Antibiotic Guardians (www.antibioticguardian.com)
Personnel required	Ensure sufficient numbers of staff/students, all familiar with overall aims and activities and informed about key messages	**Welcome table: one person;** **AMR discussion: 2–3 people** What is AMR [27]? Why is it important? **Swabbing table: 2–3 people** **Biofilm discussion and activity: 3–4 people** What is a biofilm? How common are they/where are they? What do they look like? Why is AMR important in biofilms? - medical implants; - drug-resistant biofilms; - transmission of resistance through biofilm? Build biofilms with Bunch’ems **Floating support staff: 2 people** **Observers: 2 people** [8] **Estimate 12 personnel required**
Evaluation	Quantitative and qualitative assessment regarding achievement of aims	Survey of ‘do you know what a biofilm is?’ (yes/no) Survey of understanding/information about AMR Number of agar plates used Number of visitors to Flickr (presenting images of plates post-incubation) Qualitative observation of discussion/questions/activities Photographs/images of biofilms being built/Twitter hashtag usage #buildabiofilm Number of Antibiotic Guardian sign-ups/leaflets taken
Actions	Preparation in advance of the event	Health and safety documents/risk assessment List of staff/volunteers’ names Ethical approval for survey, agar plates/swabs and observers Produce pop-up Develop yes/no test for prior knowledge of biofilms Provide substrata/backdrop for Bunch’ems (e.g., giant microscope slides, other surfaces) Practice building biofilms Produce biofilm slideshow Develop Q&A for iPad Identify key messages and provide them to the team (laminated) Sheet for observers Antibiotic Guardian information
Logistics		Arrival time and set up Staff rota Refreshments Transport of agar plates and other equipment Briefing pre-event, with key messages Debriefing post-event, fix date/time
